# Haloperidol Versus Atypical Antipsychotics for Treating Delirium in Intensive Care Unit Patients: A Systematic Review

**DOI:** 10.7759/cureus.30641

**Published:** 2022-10-24

**Authors:** Akhil Sadhu, Carla Valencia, Hameeda Fatima, Ijeoma Nwankwo, Mahvish Anam, Shrinkhala Maharjan, Zainab Amjad, Abdelrahman Abaza, Advait M Vasavada, Safeera Khan

**Affiliations:** 1 Family Medicine, California Institute of Behavioral Neurosciences & Psychology, Fairfield, USA; 2 Internal Medicine, California Institute of Behavioral Neurosciences & Psychology, Fairfield, USA; 3 Research, California Institute of Behavioral Neurosciences & Psychology, Fairfield, USA; 4 Internal Medicine, Dr. Ruth K. M. Pfau Civil Hospital Karachi, Karachi, PAK; 5 Internal Medicine, King Faisal University, Al-Ahsa, SAU; 6 Pathology, California Institute of Behavioral Neurosciences & Psychology, Fairfield, USA; 7 Medicine, Meghji Pethraj Shah Medical College, Jamnagar, IND; 8 Emergency Medicine, All India Institute of Medical Sciences, New Delhi, IND; 9 Plastic Surgery, All India Institute of Medical Sciences, New Delhi, IND

**Keywords:** pharmacology, intensive care unit stay, delirium, atypicalantipsychotics, haloperidol

## Abstract

Delirium is a severe and variable neuropsychiatric illness that causes cognitive and behavioral problems as well as abrupt impairment in consciousness and focus. Due to the complex, dynamic, and multifaceted interactions between several risk factors, the etiology of delirium is unclear. Although its efficacy has not been thoroughly studied, haloperidol, a common antipsychotic medicine, is frequently used to prevent delirium in critically ill patients. When evaluating the atypical antipsychotic response rates for treating delirium, only a few trials have taken age into account.

Articles were searched for from PubMed, PubMed Central (PMC), and Science Direct, and reviewed systematically. A complete 225 articles were identified after applying the search strategy to these databases. Out of these, 12 were finalized for review. We reviewed the efficacy and safety of haloperidol with atypical antipsychotics for treating delirium in intensive care unit patients.

## Introduction and background

Delirium is a common, dangerous, and undertreated acute disease of attention and cognition in elderly adults. A complete cognitive assessment and history of onset of symptoms are required for diagnosis. As a neuropsychiatric disorder with an underlying cause, this has been considered a diagnosis reserved for hospital settings [1]. Early coronavirus disease studies have estimated rates of delirium at 25% in hospitalized and 65% in intensive care unit patients [2]. Early identification of patients with delirium is critical for coronavirus disease patients because the occurrence of delirium is also an early symptom of worsening respiratory failure or of infectious spread to the central nervous system mediated by potential neuroinvasive mechanisms of the coronavirus [3].

The pathogenesis of delirium has been attributed to neuroinflammation, an abnormal stress response, neurotransmitter imbalances, and changes in neural networks. When a patient is suffering from a serious disease, delirium typically develops in more susceptible people (such as the elderly and cognitively impaired). Delirium in the intensive care unit is also associated with cognitive issues such as memory and attention loss, difficulties focusing, and decreased awareness. Adulthood, drunkenness, vision/hearing impairment, and, for critically ill patients, the use of restraints, prolonged discomfort, and several drugs are risk factors for delirium [4,5]. 

Patients with several risk factors seem to be sensitive to even small triggering insults, whereas those without such risk factors might only experience delirium after a serious insult (e.g., sepsis). The exact etiology of delirium is unknown; however, evidence points to the possibility that a number of biochemical networks may interact to produce the illness. A relative cholinergic deficiency and/or dopamine excess are most strongly supported by recent data, while other neurotransmitters are implicated. The most common pharmacologic options are neuroleptics and benzodiazepines; however, the use of benzodiazepines in delirium patients is frequently disputed because, to our knowledge, no randomized research has ever evaluated the impact of a benzodiazepine and a placebo on any delirium outcomes. Nevertheless, some medical professionals feel that benzodiazepines should be avoided when treating delirium because lorazepam was found to be less effective than haloperidol and chlorpromazine and was also found to be responsible for a large number of unfavorable side effects in a small randomized clinical trial. Preventing delirium is an important strategy for reducing its frequency and complications. The mainstay of treatment, antipsychotics, have demonstrated efficacy in managing delirium symptoms and enhancing cognition. Since haloperidol has fewer anticholinergic adverse effects than other antipsychotics, it is regarded as a first-line treatment. Risperidone, olanzapine, and quetiapine have the greatest data supporting their usage among the other atypical antipsychotics examined in delirium. Other medications (aripiprazole, flumazenil) have been considered, but there is not enough information [6-8].

## Review

Methods

We included studies when delirium was diagnosed in intensive care unit conditions. We used databases like Pubmed, PubMed Central (PMC), and Science Direct to look for articles using keywords and the Medical Subject Headings (MeSH) strategy. The entire study is conducted in accordance with the Preferred Reporting Items for Systematic Review and Meta-Analyses (PRISMA) 2020 criteria, which were created to make systematic review reporting more transparent and thorough. We hope that uptake of the PRISMA 2020 statement will lead to a more transparent and accurate reporting of systematic reviews [10].

Search study

The following MeSH technique was created for PubMed using Boolean AND OR ("Delirium/drug therapy"[Majr] OR "Delirium/prevention and control"[Majr] OR "Delirium/therapy"[Majr]), ("Haloperidol/administration and dosage[Majr] OR "Haloperidol/adverse effects"[Majr] OR "Haloperidol/therapeutic use"[Majr] OR "Haloperidol/toxicity"[Majr]), ("Dopamine Antagonists/administration and dosage"[Majr] OR "Dopamine Antagonists/adverse effects"[Majr] OR "Dopamine Antagonists/therapeutic use"[Majr] OR "Dopamine Antagonists/toxicity"[Majr] "delirium"[MeSH Terms] OR "delirium"[All Fields] OR "delirium's"[All Fields] OR "deliriums"[All Fields]"intensive care units"[MeSH Terms] OR ("intensive"[All Fields] AND "care"[All Fields] AND "units"[All Fields]) OR "intensive care units"[All Fields] OR "icu"[All Fields]).

The articles were screened mostly relevant to the search question and selected according to inclusion/exclusion criteria. The inclusion and exclusion criteria are summarized in Table 1.

**Table 1:** The inclusion and exclusion criteria

Inclusion criteria	Exclusion criteria
Papers published in the past 10 years	Papers are written before 10 papers
Papers with the geriatric population	Papers with the pediatric population
Papers related to the question	Papers not related to the question
Research papers were done on humans	Research papers were done on animals

All articles were combined in an Excel sheet (Microsoft, Redmond, Washington) for duplicate removal. The records were initially reviewed based on the titles and abstracts, and irrelevant articles were excluded, followed by studying the full-text articles.

Results

We looked through PubMed and Science Direct, and a total of 225 papers were found after 193 studies from PubMed and 32 from Science Direct were combined. Using EndNote (Clarivate, London, United Kingdom), 57 duplicate articles are eliminated, leaving 168 articles in total for screening. Based on their titles and abstracts, those articles were chosen for screening. Title and abstract screening eliminated 104 records. Thirty-seven publications in all were evaluated for eligibility, and 25 were eliminated for the reasons listed in the PRISMA chart. After screening, 12 articles dealt with comparing the safety and efficacy of haloperidol vs. atypical antipsychotics for treating delirium in intensive care unit patients. We describe the quality appraisal for studies included in this systemic review in Table 2.

**Table 2:** Summary of randomized controlled trials using the Cochrane assessment tool

Cochrane appraisal	Year of study	Random sequence generation	Allocation concealment	Blinding of participants & personnel	Blinding of outcome assessment	Incomplete outcome data	Selective reporting
Slooter et al. [4]	2019	Yes	Yes	No	No	No	Yes
Girard et al. [8]	2018	Unclear	Yes	No	No	No	No
Duprey et al. [9]	2021	Yes	No	No	Unclear	No	No
Chen et al. [11]	2020	Yes	Yes	No	No	No	Unclear
Lin et al. [12]	2020	Unclear	Yes	No	No	No	Yes
Rivière et al. [13]	2019	Yes	Yes	Yes	Unclear	No	No
Page et al. [15]	2013	Unclear	Unclear	Yes	Yes	Yes	Unclear

Each clinical study examined qualitative investigations, therapies, and results in a unique way. Table 3 is a tabular summary of the clinical studies.

**Table 3:** Summary of clinical trials

Author & year of publication	Intervention studied	Type of study	No. of patients	Conclusion
Duprey et al., 2021 [9]	Haloperidol vs. placebo	Randomized controlled trial	1495	Haloperidol therapy for incident delirium and related symptoms may be linked to dose-dependent survival.
Girard et al., 2018 [8]	Haloperidol, ziprasidone vs. placebo	Randomized controlled trial	1193	This intervention significantly did not alter the duration of delirium.
Page et al., 2013 [15]	Haloperidol vs. placebo	Randomized controlled trial	142	Intravenous haloperidol should only be used to treat acute agitation temporarily.
Grover et al., 2011 [16]	Olanzapine, risperidone vs. haloperidol	Randomized controlled trial	64	When treating delirium, risperidone and olanzapine are just as effective as haloperidol.

**Figure 1:**
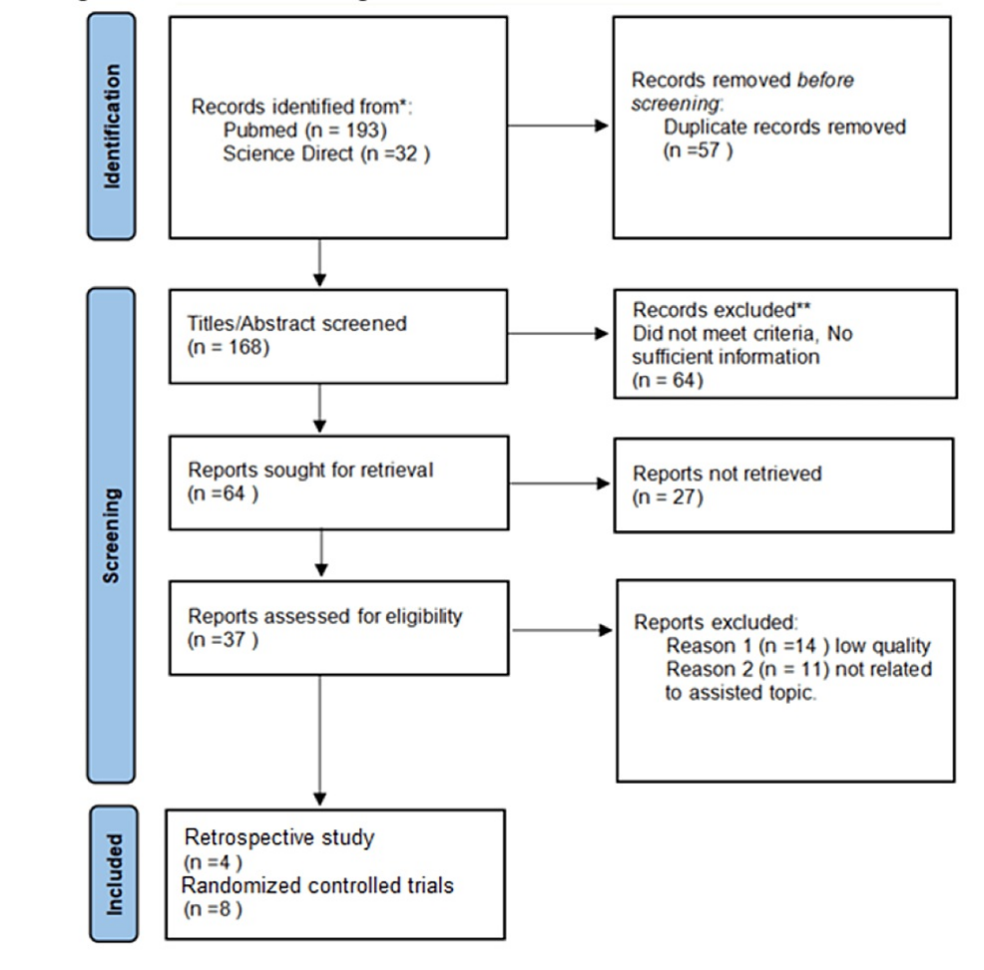
PRISMA flow diagram Adapted from source: Page et al. [10] PRISMA - Preferred Reporting Items for Systematic Review and Meta-Analyses

A PRISMA flow chart presenting the selection of articles is shown in Figure 1.

Discussion

We conducted a systemic review to examine the evidence on the efficacy and tolerability of haloperidol vs. atypical antipsychotics in the treatment of delirium. Despite two modest randomized controlled trials finding no indication that haloperidol causes delirium to last longer in the intensive care unit than a placebo, the common antipsychotic drug haloperidol is still used to treat delirium in the ICU. Haloperidol's safety profile shows no association with extrapyramidal symptoms, prolonged QT, or negative side effects [6].

One thousand four hundred ninety-five critically ill people with delirium were admitted to an intensive care unit, with an expected stay of more than two days. Patients received preventive haloperidol or a placebo for up to 28 days until the onset of delirium, death, or discharge from the intensive care unit. If delirium occurred, treatment with IV haloperidol 2mg was administered at medical discretion. Patients were evaluated for delirium and coma for 28 days. For 28 days, patients were monitored for delirium and coma. In order to control for research arm, delirium and coma days, age, mechanical ventilation, and length of intensive care unit stay, Cox hazards models for 28-day and 90-day mortality were created. Among the 1,495 patients, 542 (36%) advanced delirium within 28 days (with delirium for a median of four days [IQR: two to seven days]). Haloperidol (2.1 mg [1.0-3.8 mg] daily) was administered as a treatment to 477 out of 542 people (88%) for a total of six days. Haloperidol treatment dosages of one milligram per day were linked to lower mortality at 28 days (hazard ratio, 0.93; 95% confidence interval, 0.91-0.95) and 90 days (hazard ratio, 0.97; 95% confidence interval, 0.96-0.98) [9].

Three thousand thirty-four patients were included in eight randomized control trials that used both including and excluding criteria. Patients receiving haloperidol prophylaxis and those receiving a placebo did not substantially vary in terms of the incidence of delirium, according to pooled studies (relative risk (RR) = 0.90, 95% confidence interval (CI) = 0.70 to 1.15), inconclusive. Notably, compared with the control group, the use of haloperidol significantly decreased the duration of delirium (Mean difference (MD) −0.94; 95% CI −1.82 to −0.06 days), with marked heterogeneity. Additionally, the duration of mechanical ventilation, the length of the intensive care unit stay, the length of the hospital stay, and mortality are no longer significantly impacted by haloperidol prophylaxis [11]. Haloperidol did not significantly lower the prevalence of delirium in all patients in the intensive care unit when compared to placebo (RR, 0.83; 95% CI, 0.62-1.10, p = 0.20). However, it could reduce the incidence of delirium in postoperative patients who are admitted to an ICU (RR, 0.63; 95% CI, 0.47-0.86, p = 0.004) [12].

Comparability with atypical antipsychotics

Atypical antipsychotics (AAPs) are becoming more and more relevant, despite the fact that haloperidol is still the most widely prescribed medication for the treatment of delirium. The evidence doesn't seem to be sufficiently limited to support the efficacy of olanzapine, despite the fact that it has been shown to be equally safe and effective as haloperidol in a number of controlled trials. Additionally, uncontrolled studies have also shown positive benefits, making olanzapine a safe option to haloperidol. Only quetiapine, risperidone, and ziprasidone have additionally been investigated in randomized control trial designs among the other atypical antipsychotics described. Small-scale randomized control trials suggest quetiapine to be an effective and safe alternative to haloperidol, as do the trials on risperidone, barring one, the important largest randomized control trial, which reported poorer outcomes compared to placebo, with the possibility of survival being decreased after haloperidol and risperidone. These three and additional atypical antipsychotics, including aripiprazole, paliperidone, and perospirone, were the subjects of numerous open trials, which were analyzed and came to the same conclusion that the medications were both efficient and safe for treating delirium [13]. There was no proof that either ziprasidone or haloperidol contributed to a reduction in the length of delirium or coma in this double-blind, randomized, placebo-controlled trial of intravenous antipsychotic medicines for the treatment of delirium in the intensive care unit. Patients who received up to 20mg of haloperidol or up to 40mg of ziprasidone daily, or those who received a placebo, had comparable outcomes in terms of survival and lengths of stay in the critical care unit and hospital [3].

Twelve randomized control trials and 22 open trials were taken into consideration. Olanzapine and quetiapine have been shown to be effective in placebo-controlled trials, despite the general dearth of large-scale randomized control trials. In a very recent and enormous randomized control trial in elderly patients, risperidone and/or haloperidol were linked to a considerably worse outcome than placebo. Even though the atypical antipsychotics olanzapine, quetiapine, and risperidone are thought to be as efficacious as haloperidol in the current comparative trials, treatment with these drugs is linked to a lower incidence of extrapyramidal symptoms. Now, it has been established that ziprasidone is no longer effective [13].

Quetiapine dosage is titrated to get the desired impact while treating delirium in critically ill patients. The purpose of this study was to evaluate quetiapine's safety for this use. One hundred fifty-four critically ill patients, who were identified through a review of their medical records, were initiated on quetiapine for the treatment of delirium and followed for QTc prolongation. The median average daily dose was 150mg (79-234), and the median max dose was 225mg (100-350). The overall range was 25-800mg daily. The time to peak dose was three days (one to eight). Patients with QTc prolongation were significantly older (age 54 ± 11 v.s 45 ± 17 years, p = 0.002) and with higher baseline QTc (454 ± 33 vs. 442 ± 30 (p = 0.045)). Multivariant analysis revealed only dose as a major factor (OR = 1.006 (1.003-1.009), p < 0.001) [14]. These results do not support the hypothesis that haloperidol shortens delirium in individuals with serious illnesses. Although haloperidol is frequently used in this patient population safely, at this time, the use of intravenous haloperidol should be limited to the short-term treatment of acute agitation [15].

There is no documented evidence that treating adult intensive care unit patients with haloperidol shortens their delirium occurrence or duration. Despite being frequently used in clinical practice to prevent and treat delirium in the critically ill, it has also been employed as a good quality improvement intervention and as part of routine sedation practices [15].

Limitations

Our search was limited to studies within the English language and excluded grey literature. There have been limited studies and articles available on the efficacy and safety of haloperidol vs. atypical antipsychotics. Access to full-text articles was low due to most being articles that require payment for access. The use of antipsychotic medications to treat delirium in patients in intensive care units is still controversial, according to several studies.

## Conclusions

Our systemic review brings how the efficacy and safety of haloperidol compared with atypical antipsychotics used for treating delirium in the intensive care unit. Olanzapine, risperidone, and quetiapine appear to be adequate alternatives to haloperidol in patients who are susceptible to extrapyramidal symptoms, despite the fact that haloperidol may be used safely in this population, given the current evidence of efficacy and tolerability of atypical antipsychotics in the treatment of delirium is limited. Appropriate drug therapy should be considered a part of systematic approaches to delirium treatment and prevention. There is a necessity for randomized, double-blinded controlled trials investigating drug management of varied aspects of delirium, including dose-ranging studies and optimal duration of therapy.
